# *Legionella* Research: Still Many Miles to Go

**DOI:** 10.3390/biom13050775

**Published:** 2023-04-29

**Authors:** Yury Belyi

**Affiliations:** Gamaleya Research Centre for Epidemiology and Microbiology, Moscow 123098, Russia; belyi@gamaleya.org; Tel.: +7-499-193-55-30

*Legionella* is a widespread Gram-negative bacterium occurring in water reservoirs and soils. Its life cycle is dependent on intracellular proliferation in free-living unicellular organisms (amoebae and ciliated protozoa) in close association with other inhabitants of ecological biosystems. In humans, the microorganism multiplies in phagocytic cells and is an infectious agent of legionellosis. This aerosol-borne disease manifests either as severe pneumonia, termed “Legionnaires’ disease”, or as relatively mild nonpneumonic “Pontiac fever” [1,2].

The ability of *Legionella* to infect eukaryotes depends upon the Dot/Icm type 4B secretion system (T4BSS) to deliver a set of highly specific *Legionella*-encoded effector proteins into the targeted cells. The bacteria manipulate various host processes to support bacterial replication in *Legionella*-containing vacuoles and the egression of the pathogen from the cell in later stages of the life cycle [3,4].

Due to their important roles in bacterial virulence, T4BSS effectors have attracted considerable attention from researchers. Accordingly, two manuscripts from this Special Issue address these proteins. A review paper discussed the role of glycosyltransferases in *Legionella* virulence mechanisms [5]. Investigations of these enzymes were carried out during the last two decades, starting from the discovery of the first toxic glucosyltransferase Lgt1 in 2003, proceeding to the description of several other enzymes—Lgt2, Lgt3, SetA, SidI, and LtpM. These studies resulted in the description of an expanding group of unique *Legionella* proteins with diverse targets, structures, enzymatic mechanisms, regulatory mechanisms, and biological effects. In another manuscript, Kevin Voth et al. [6] were successful in solving a crystal structure of MavL, an effector initially identified during a screen for the translocated substrates of T4BSS [7]. The protein has remained enigmatic thus far. As shown in the current investigation, MavL exhibited distinct structural features of ADP-ribose-binding proteins and binds ADP-ribose but lacked evident in vitro glycohydrolase activity. The data presented in the manuscript suggested that the effector functioned as “an ADP-ribose reader”, participating in ubiquitination pathways during *Legionella* infection by sensing ADP-ribose modifications in the target cell.

Biofilm formation by *Legionella* represents a smart strategy utilized by the pathogen to survive in harsh environments and is an underestimated and, hence, poorly studied phenomenon. The study by Courtney Marin et al. is, therefore, of special importance [8]. The authors were able to identify a gene, termed *bffA*, whose inactivation increased the growth rate of single *L. pneumophila* colonies on agar media, enhanced bacterial uptake by *Acanthamoeba castellanii*, decreased flagellar motility at 37 °C, and enhanced biofilm formation. The provided data suggested a link between *bffA* function and the quorum sensing (QS) circuits in *Legionella*. Interestingly, the product of the gene did not contain typical signatures of the c-di-GMP metabolizing enzymes known to participate in QS reactions, and the precise type of regulatory network, in which the *bffA* product functions, remains to be determined.

It was established that some housekeeping proteins produced by pathogenic bacteria could accomplish virulence-associated functions [9]. Chaperons were among such molecules. Accordingly, the 60-kDa GroEL-related chaperonin of *L. pneumophila* (HtpB) appeared to play many folding-independent roles in *Legionella* virulence [10]. Karla N. Valenzuela-Valderas et al. [11] compared the primary structures of GroEL and HtpB and were successful in demonstrating that at least 10 amino acid residues were important for the interaction of HtpB with the eukaryotic proteasome-related protein ECM29. Using Evolutionary Trace Analysis, the authors identified and mutated amino acid residues potentially involved in the protein-folding-independent functions of HtpB. Through various methods, the authors showed that the binding of mutated HtpB molecules to hECM29 was significantly impaired. Moreover, the engineering of the corresponding mutations in *E. coli* GroEL resulted in a weak but significant GroEL-ECM29 interaction.

The innate immunity of macroorganisms plays an essential role in detecting and eradicating intruding pathogens by specifically sensing conserved pathogen-associated molecular patterns (PAMPs) [12]. Bacterial flagellin represents one such PAMP and is detected by the membrane-bound eukaryotic TLR5 receptor. Lina Scheithauer et al. [13] demonstrated that the metalloproteinase ProA produced by *L. pneumophila* was able to degrade the monomers of bacterial flagellin and thus antagonized the flagellin-mediated TLR5 stimulation and the subsequent activation of the proinflammatory NF-κB pathway. These data show that *Legionella* has developed a strategy for counteracting mammalian host immunity pathways, thus promoting immune evasion of the pathogen, by using ProA.

More than 45 years have passed since the identification of *L. pneumophila* as an infectious agent for a serious human disease. The organism has attracted immense attention since the very beginning of this story, and this interest has not diminished over time. Papers published in the current issue clearly demonstrate the modern and divergent character of this research, elucidating the amazing strategies used by both the pathogen and the host in their struggles to live.
